# Vaccination programs for older adults in an era of demographic change

**DOI:** 10.1007/s41999-018-0040-8

**Published:** 2018-03-19

**Authors:** T. Mark Doherty, Mark P. Connolly, Giuseppe Del Giudice, Johan Flamaing, Jorg J. Goronzy, Beatrix Grubeck-Loebenstein, Paul-Henri Lambert, Stefania Maggi, Janet E. McElhaney, Hideaki Nagai, William Schaffner, Ruprecht Schmidt-Ott, Edward Walsh, Alberta Di Pasquale

**Affiliations:** 1 GSK, Wavre, Belgium; 2 Global Market Access Solutions, St-Prex, Switzerland; 3 Unit of PharmacoEpidemiology and PharmacoEconomics, Department of Pharmacy, University of Groningen, Groningen, The Netherlands; 4 GSK, Siena, Italy; 5 Department of Geriatric Medicine, University Hospitals Leuven, Leuven, Belgium; 6 Division of Gerontology and Geriatrics, KU Leuven, Leuven, Belgium; 7 Division of Immunology and Rheumatology, Department of Medicine, Stanford University, Stanford, CA USA; 8 Institute for Biomedical Aging Research, University of Innsbruck, Innsbruck, Austria; 9 Center of Vaccinology, University of Geneva, Geneva, Switzerland; 10 CNR Institute of Neuroscience, Aging Branch, Padua, Italy; 11 Health Sciences North Research Institute, Sudbury, ON Canada; 12 Center for Pulmonary Diseases, National Hospital Organization Tokyo National Hospital, Tokyo, Japan; 13 Vanderbilt University School of Medicine, Nashville, TN USA; 14 Department of Medicine, School of Medicine and Dentistry, University of Rochester, Rochester, NY USA

**Keywords:** Demographic change, Healthy aging, Vaccines, Vaccination programs

## Abstract

**Objectives:**

Populations are aging worldwide. This paper summarizes some of the challenges and opportunities due to the increasing burden of infectious diseases in an aging population.

**Results:**

Older adults typically suffer elevated morbidity from infectious disease, leading to increased demand for healthcare resources and higher healthcare costs. Preventive medicine, including vaccination can potentially play a major role in preserving the health and independence of older adults. However, this potential of widespread vaccination is rarely realized. Here, we give a brief overview of the problem, discuss concrete obstacles and the potential for expanded vaccination programs to promote healthy aging.

**Conclusion:**

The increasing healthcare burden of infectious diseases expected in aging populations could, to a large extent, be reduced by achieving higher vaccination coverage among older adults. Vaccination can thus contribute to healthy aging, alongside healthy diet and physical exercise. The available evidence indicates that dedicated programs can achieve substantial improvements in vaccination coverage among older adults, but more research is required to assess the generalizability of the results achieved by specific interventions (see Additional file 1).

**Electronic supplementary material:**

The online version of this article (10.1007/s41999-018-0040-8) contains supplementary material, which is available to authorized users.

## Introduction

Worldwide, populations are aging due to ever increasing life expectancy and decreasing birth rates. The United Nations estimates that 15% of the world’s population will be over the age of 60 years by 2025 and that this proportion will rise to well over 20% by 2050 [1]. In Europe as well as Japan, the proportion of people aged 65 years or older will double from 2010 to 2060 and the proportion of people aged 65 years or older relative to the population of working age (15–64 years) is expected to double by 2060.

This demographic development is already starting to put considerable strain on public finances in countries with state-financed pensions and healthcare systems and the effects can only be expected to increase [2, 3]. Without substantial (and politically difficult) changes in policy, the tax base will diminish because of the fall in the proportion of working age (and tax-paying) adults while pension expenses will grow, both because of continued increases in life expectancy and the sheer growth in the number of people eligible for pensions. Furthermore, it is expected that healthcare expenditures will increase substantially, as individual need for healthcare services rises markedly with advancing age.

Only recently have the implications and the dimensions of the coming problems been more widely recognized, leading to various strategies being debated. These various measures are aimed at keeping older adults economically and socially active longer than has been common in prior generations on the one hand—the concept of “adding life to years”—and on the other to delay as much as possible the inevitable age-related increase in healthcare utilization—a concept designated as “healthy aging” [4].

## Discussion

### Healthy aging

The World Health Organization has recently defined the concept of healthy aging as “the process of developing and maintaining the functional ability that enables well-being in older age” [4]. Healthy aging is obviously a laudable objective on its own to improve welfare and quality of life (QoL), but it is also specifically recognized as necessary to counter the anticipated surge in healthcare costs consequent on the demographic change underway.

**Fig. 1:**
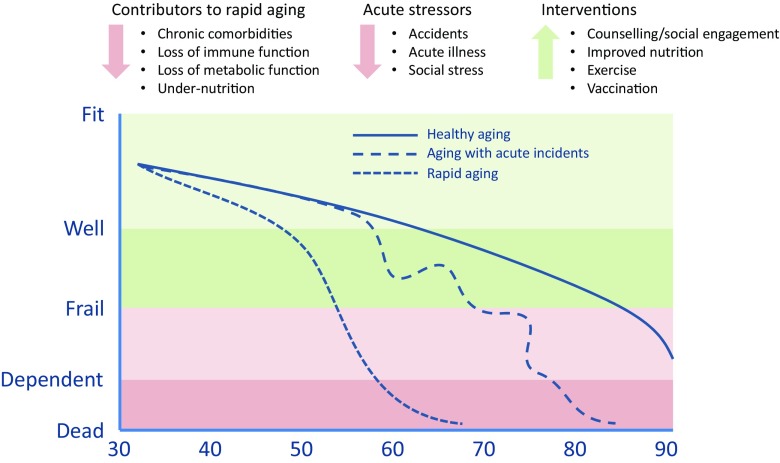
Healthy vs premature aging. Typically, aging is associated with a decline in physical capability, during adulthood, from being fit and physically active to being well (that is, without obvious physical incapacity, but with reduced physical activity). In most adults, the later phases of life are characterized by frailty and disability. Disability is simply enough defined as the inability to perform basic day-to-day functions without assistance. Frailty is more difficult to define, but is often identified as meeting 3 or more of the following clinical criteria: a low level of physical activity, exhaustion or low energy, muscle weakness, slowness, and unintentional weight loss. The underlying etiology of frailty is poorly understood, but includes comorbidities such as diabetes, obesity and respiratory illnesses. Immune dysfunction also seems to be a key factor in two ways. First, through chronic inflammation, mitochondrial dysfunction and cellular senescence that may degrade the endocrine, hematologic and musculoskeletal systems, and secondly, by increasing susceptibility to infections, which can further harm these physiologic systems. The ideal of healthy aging (living with as little time spent in frailty or disability) is contrasted to rapid aging in this simple schematic. In reality, however, aging for most people is a nonlinear process. Acute incidents such as physical trauma from falls, social stressors such as divorce or death of a spouse, or acute illness (for example, from cardiac disease or infection) can trigger sudden losses of capacity, which in older individuals, become increasingly difficult to recover from. This places a priority on intervention to prevent those acute incidents to prevent frailty/disability and maintain quality of life

Traditional stereotypes are no longer applicable to the current population of older adults, who are often healthier and more active than prior generations and the focus today is not on chronological age but on functional ability and independence [4]. In particular, there is consensus that we need to delay the onset of “frailty”, a common clinical syndrome in older adults that carries an increased risk for poor health outcomes including disability, hospitalization and mortality [5, 6] (Fig. 1). Development of frailty is often associated with a decreased ability to respond to immune stimuli, so-called immunosenescence (see Text Box 1). Lower immune responses in older adults correlate with higher susceptibility to infectious diseases and a higher risk of hospitalization or serious outcomes than in a younger person, further complicated by the higher prevalence of comorbidities common in older adults [7].

For example, infections of the lower respiratory tract are now the fourth most frequent cause of death in developed countries, with approximately 75% of cases occurring in adults aged 60 years and older [8, 9]. As another example, in the United States it is estimated that 40,000–80,000 people die annually from vaccine-preventable diseases (VPDs), while hundreds of thousands more are hospitalized [1, 10]. The majority of cases and 99% of the deaths from VPDs are in older adults [11]. Given these numbers and existing trends in disease incidence, infectious diseases represent a major barrier to healthy aging and the burden of infectious diseases in adults over age 60 years is coming to represent a significant and increasing proportion of healthcare expenditures [12].

### Text box 1. Immunological frailty

Frailty is a syndrome that covers the functional ability of an individual in both mental and physical spheres, and increasing frailty is associated with decreased capacity in these areas and an increased risk of mortality. Frailty can be viewed as either an accumulation of comorbidities, or as a loss of the ability to respond effectively to environmental stressors [13, 14]. Regardless of definition, there is consensus that increasing frailty is associated with an increased risk for further physiological decline, suggesting a causal linkage [13].

While aging is clearly linked to frailty, both terms are not synonymous. Some individuals become clinically frail by the age of 70 years, while in others this may not happen until 90 years of age [14]. Frailty also seems to be tightly linked to loss of immunocompetence and greater susceptibility to infection (a combination referred to as immunosenescence), to the extent that immune dysregulation and chronic inflammation have been suggested as primary drivers of frailty [15]. Consistent with this, biomarkers typically associated with increased inflammation appear to be predictive of frailty, mortality and elevated disease risk [16, 17]. Since frailty appears to be a better predictor of disability, institutionalization and mortality than chronological age, the implications for both healthcare costs and personal QoL, if we can understand how to delay the onset of frailty, are enormous.

The elevated risk of infection and disease in older adults, and especially in the frail elderly, correlates with a decreased ability to respond to vaccination. It is also associated with a decreased pool of naive T cells, a relative increase in the proportion of memory T cells and an increased proportion of CD8+ T cells [18]. While it reaches pathological levels in the frail elderly, immunosenescence can be viewed as the culmination of a process that develops throughout life, in response to continuous immunological stimulation: in some cases, decreased immune responses to vaccination can be seen even in young adults compared to children [19, 20].

### Vaccination—a tool for healthy aging

Increasing vaccination coverage of older adults against VPDs can be expected to promote healthy aging. The VPDs particularly relevant for older adults currently include seasonal influenza, invasive pneumococcal diseases, pneumonia, herpes zoster (shingles), meningococcal diseases, pertussis, diphtheria, tetanus and hepatitis [21]. Numerous studies have been carried out to assess vaccination of older adults against these VPDs in terms of avoided cases and savings in healthcare resources and costs (e.g., [10, 22–24]). As just one example, a Europe-wide study showed that vaccination coverage of 75% of adults over 65 against seasonal influenza could result in 1.6–2.1 million cases prevented, 25,000–37,000 influenza-related deaths avoided and savings of healthcare costs amounting to €153–219 million annually [22]. This is probably only the tip of the iceberg as there is substantial additional morbidity associated with infectious diseases in terms of serious sequelae (see Text Box 2). Even when vaccination is not 100% effective in preventing infection with a pathogen, it may still attenuate the course and severity of a disease [25, 26].

Despite the availability of effective and well-tolerated vaccines against these diseases, many countries struggle to reach recommended coverage levels even when vaccination is supported by national programs. The reasons include lack of knowledge, poor infrastructure for adult vaccination or perceptions that the benefits of vaccination of older adults may not justify the costs (see Text Box 3).

### Text box 2. The hidden costs of infectious disease in older adults

Cost‐effectiveness analyses of vaccination typically focus on the morbidity and mortality directly attributable to a specific pathogen and the percentage of this burden of disease that can be prevented by a vaccination program. However, in older adults, particularly those of advanced age, comorbidities such as cardiovascular disease, type‐2 diabetes, chronic obstructive pulmonary disease and renal or hepatic dysfunction are common, and these can be negatively impacted by infectious diseases [27]. In addition, the course of disease in older adults is typically more severe, the period of recovery more prolonged, and the risk of complications higher. As one example, herpes zoster (HZ) in younger adults is uncommon, typically transient and usually resolves without serious complications. In older individuals however, not only does the incidence of HZ increase significantly but so does the risk of complications such as postherpetic neuralgia, which in some cases can cause chronic, debilitating pain lasting for months or even years [28]. Older HZ patients also have an excess risk of stroke amounting to 30% in the year after HZ onset [29]. Combined, these factors represent a significant risk to the patient’s QoL and their ability to continue working or living independently [30].

Similar data are available for other VPDs. One study of 36,636 outpatients aged ≥ 65 years with a chronic illness indicated that vaccination against influenza and pneumococcus reduced the risk of ischemic stroke and acute myocardial infarction by approximately a third. Compared with unvaccinated individuals, the vaccinated persons had a substantially reduced risk of death and reduced risk of coronary and intensive care admissions in the year following vaccination [31]. Indeed, the commonest causes of severe disability in older adults including strokes, congestive heart failure, pneumonia, ischemic heart disease, cancer, and hip fracture, have all been linked to influenza [18]. In the US, 90% of the estimated 30–40,000 deaths linked to influenza occur in older adults and are related to cardiovascular and pulmonary complications [32].

The costs resulting from loss of employment, loss of independence and need for rehabilitation or inpatient care due to chronic disease arising subsequent to infection can be substantial. These risks can potentially be significantly reduced by vaccination, but are typically not considered in analyses of vaccine cost‐effectiveness [33].

### Text box 3. Challenges for vaccination in older adults

The most immediate challenge to vaccination in older adults is the decreased immune response associated with aging. This is characterized not just by decreased specific antibody titers generated by vaccination, but also by a more rapid decline in titers, suggesting rapid loss of immune memory [34]. The mechanisms involved are not yet fully defined, but do not appear to involve the loss of immune cells capable of recognizing the response [35]. Instead, it may reflect a change in the balance of existing immune memory and a relative decline in the proportion and types of memory T cells [36, 37]. If correct, this could explain the difficulty of generating durable memory responses with vaccines containing higher doses of antigen. This may simply boost an existing population of differentiated antigen‐specific cells which are already dysfunctional with regard to immune memory [36]. However, the observation that adjuvanted vaccines can generate stronger immune responses that apparently persist for longer periods of time, even in individuals aged 70–90 years, suggests that, given the correct stimuli, functional memory responses can be generated de novo from the existing pool of T cells [38–41].

Improved vaccine efficacy has consequences beyond those expected from a reduction in disease incidence in vaccinated individuals. No vaccine is effective if not used, and vaccination in older adults has been hampered by concerns among some older adults, vaccinators and public health officials that it is ineffective or too short‐lived to be worth pursuing—in other words that the benefit/risk ratio is low [42]. Vaccines that can demonstrate enhanced efficacy and durable protection in older adults may therefore be key to persuading the population to request them and public health systems to deliver them.

### Immunosenescence

Human aging is characterized by a chronic, low-grade inflammation, a phenomenon termed “inflammaging”, which is a highly significant risk factor for morbidity, frailty and mortality in older adults as most, if not all, age-related diseases share an inflammatory pathogenesis [43]. This low-grade inflammation not only accelerates tissue degeneration and wasting, but also influences adaptive immune responses which are at the core of generation of immune memory and, therefore, immunization.

Activation of the innate immune system resulting in the production of cytokines during aging can be caused by waning control of latent infections, less effective physical barriers (more permeable skin, defective mucosal barriers in gut and respiratory systems) or increase in overall tissue damage [44, 45]. In addition, age-associated intrinsic defects in innate immune cells as well as accumulation and activation of non-immune cells such as adipocytes contribute to inflammaging.

The initiation of a T-cell response to a vaccine requires activation of dendritic cells that present antigenic fragment peptides derived from the vaccine to T cells. This process, which is the major target for the adjuvants in vaccines, appears to be disturbed in an inflammatory environment or with older dendritic cells. Understanding of the mechanisms of action of adjuvants and vaccine delivery systems and identifying those that are more effective in older individuals remain an area of active research [46, 47].

The adaptive immune response of T and B cells, the backbone of a vaccine response, is also susceptible to aging [34] and the previous decade has seen a surge in research on how and where this process is impaired in older individuals. Defects in adaptive T-cell responses already begin to become significant about the age of 50 years, in particular in individuals with comorbidities. However, at least for healthy individuals, the sizes and repertoires of antigen-specific CD4+ T cells and B cells do not appear to be decreased to a biologically relevant extent in older adults [35].

Instead, at least part of the problem seems to be the reduced ability of antigen-specific lymphocytes to survive as long-lived memory T cells [36]. It is possible that chronic infections such as cytomegalovirus may compromise antigen-specific responses and help drive the development of immunosenescence [48, 49]. Whether this defect can be overcome by better vaccines or adjuvants or requires pharmacological intervention during the development of T-cell responses after vaccination remains uncertain. In contrast to CD4+ T cells, CD8+ naive and central memory T cells, apparently the weak link in immune aging, are increasingly lost and become dysfunctional with age [37]. Depending on the infection targeted by vaccination, it might be advantageous to generate an effective CD8+ memory population earlier in life, but induction of a CD8+ T-cell response generally requires a live vaccine, whereas the inactivated or component vaccines previously developed preferentially induce CD4+ T-cell and B-cell responses.

### The economics of adult vaccination

Alongside investments in infrastructure to improve water sanitation, vaccination programs are usually considered as the most important factor in improved public health and longevity worldwide over the last century. It is increasingly recognized that proper assessments of the economic value of vaccination needs to take a broader perspective than just focusing on the clinical benefits and the avoided healthcare costs that may be attributed to prevention of a single, specific disease. The broader economic benefits to consider and attempt to quantify include improved educational attainment, productivity gains from ameliorated effects of multi-morbidity on general health and cognition, community externalities and political stability [50, 51].

Intangible benefits of vaccination considered specifically in relation to vaccination of older adults, include attenuated severity of disease in breakthrough cases [25] and reductions in complications and comorbidities. Examples are studies showing that influenza and pneumococcal vaccinations may reduce the incidence of myocardial infarction by up to 50% [52]. Another example is that herpes zoster is strongly associated with an increased risk of stroke, an excess risk which may be prevented by effective vaccines [29] (See Text Box 2).

Another consideration is that vaccination may diminish the problems related to polypharmacy in older adults with many comorbidities, which may lead to important adverse effects or lack of compliance [52]. Another intangible benefit of vaccination, which is increasingly recognized (and not limited to vaccination of older adults), is that it may reduce the use of antibiotics [53–55] and thus diminish the growing problems caused by the development of antibiotic-resistant strains of bacteria. The United Kingdom Joint Committee on Vaccination and Immunization has recently recommended that this effect be included in economic evaluations of new vaccines and vaccination programs [56].

The most widely used approach to economic evaluation of healthcare interventions (including vaccines) is cost-effectiveness or cost-utility analyses, which aim to assess the incremental benefits of the intervention relative to its incremental costs, usually taking the perspective of the healthcare system in estimating the costs. This type of economic assessment is used in many countries by healthcare authorities for making decisions about reimbursement of new treatments. For preventive interventions like vaccines, the assessments usually seek to estimate the loss of utility (QoL) avoided by the intervention and relate this to the incremental costs incurred relative to no intervention. The result is expressed as the incremental costs per quality-adjusted life-year (QALY) gained (or, more accurately, not lost), which may then be compared to a benchmark to determine whether the intervention is cost-effective or not. Commonly used benchmarks in Europe range from 20,000 to 30,000 €/QALY, but few countries have set an explicit, official limit.

A number of economic evaluations and reviews of vaccination of older adults have recently been published, for example of pneumococcal conjugate vaccines [57] and herpes zoster vaccine [58, 59]. Generally, these evaluations conclude that vaccination is cost-effective but even taking the narrow direct cost perspective there are important challenges to meet when making such assessments. Among these are heterogeneity between targeted older individuals in terms of risk of infection, response to vaccination and severity of disease, just as there may be age-related variations in duration and level of immunity induced by vaccination [60].

It is also of interest to note that, ignoring the difficult assessment and valuation of health outcomes, studies of the costs of interventions show that the costs of vaccination are lower than those of many other preventive interventions and the potential benefits substantial (see Text Box 4). A recent study estimated that the total costs of fully adhering to recommended vaccinations over the full course of life in several European countries was much lower than those of many widely used preventive measures such as taking statins to prevent cardiovascular complications [61].

### Text box 4. Vaccination programs for healthy aging

The proportion of the population over 65—and especially over 85—years of age has increased dramatically over the last half century and this trend is only expected to accelerate. While the decrease in mortality is to be celebrated, there is concern over the implication that a declining ratio of those in employment to those no longer working will place stress on government budgets, and that the greater risk of illness in older individuals will place particular stress on public health budgets [62]. Some steps have already been taken to address the first of these concerns, as seen in the many countries raising the qualifying age for retirement and pension eligibility. However, for such policies to be successful, older individuals must remain healthy enough to continue working. Additionally, while an increased demand for healthcare is unlikely to be entirely avoidable in an aging population, there is growing focus on ways of diminishing the burden of disease in older people, a concept generally called “Healthy aging”.

A key component of healthy aging strategies is the recognition that infectious disease is likely to play an increasing role in morbidity among older individuals ([63] and Text Box 2) and that vaccination is among the most cost‐effective interventions. Consequently, we have seen expansion of vaccination programs and recommendations shifting from a predominantly pediatric focus to one covering the whole lifespan, so‐called life course immunization, [64] as well as the development of vaccines specifically targeting diseases in older adults (see Text Box 3). The scale of the challenge—and the potential benefits—are dramatic. In Japan, the currently most advanced country in the demographic transition, control of infectious diseases and vaccination are among the Ministry of Health, Labour and Welfare’s top priorities [65] and the government is proposing a Healthy Aging program, which is projected to save 5 trillion yen (approx. 40 billion Euros at current exchange rates) by 2025, with almost a fifth of that coming from reduction in VPDs (primarily pneumonia) [65]. Other nations estimate potential savings of a similar magnitude. For example a recent study estimated that under-vaccination of adults costs the US 7.1 billion USD in healthcare expenditure per year [12].

### Adult vaccination: recommendations and coverage

Despite the accumulating evidence of the benefits of vaccination of older adults, vaccine uptake is generally limited and far below targets [60]. To improve this situation, it is necessary to identify the barriers to increased uptake of vaccinations among older adults and to modify these where possible.

Vaccine programs for children and younger adults have shown that great declines in the incidence of infectious diseases can be achieved if vaccination is properly implemented [25]. In the United States, coverage rates in children for most recommended vaccines reach approximately 90% [66]. European countries are more diverse, both in their recommended vaccines and the recommended schedules, but the coverage for the most common pediatric vaccines (measles, diphtheria, pertussis, tetanus, tuberculosis, polio, etc.) reaches or exceeds 90% in most EU countries [67]. These programs indicate what is needed for successful disease control by vaccination and thus demonstrate the opportunities for adult vaccination programs. Comparing current pediatric vaccination programs with what is being done to enhance vaccination of older adults also highlights important differences and the challenges that must be overcome to improve adult vaccination rates.

Where adult vaccination is recommended, there are wide divergences in what is recommended (Table 1) and in the coverage levels reached. In a recent survey of immunization policies in 31 high-income countries [68], only 12 had comprehensive adult vaccination policies, although all of them had recommendations for at least one adult vaccination (influenza, with programs in place to monitor the vaccination coverage in adults in 29 of the countries). In two countries, influenza vaccination is recommended for the entire population, whereas in the rest it was only recommended for risk groups. Despite recommendations and public funding, only one country in this study (the Netherlands) exceeded the recommended level of coverage (75%) while many reached less than 50% [68]. For the other vaccines, recommendations are most common (26–27 countries) for adult immunization against hepatitis B, pneumococcus, tetanus and diphtheria, although again, these recommendations primarily focus on risk groups and travelers [68] and coverage is generally poor [69]. Overall, the picture of adult vaccination is one of fragmented recommendations, restricted coverage [70], and significant data gaps [68].

**Table 1:** Recommended vaccinations for adults in selected high-income countries [71–78]

Vaccine	Recommended vaccination	Country
Diphtheria	No national recommendation	Croatia, Czech Republic, Denmark, Hungary, Iceland, Ireland, Japan, Republic of Korea, Malta, Netherlands, Norway, Romania, United Kingdom
	Single booster	Poland (in adulthood), Spain (at age 65)
	Adult, every 10 years	Austria^a^, Belgium, Bulgaria, Canada, Cyprus, Estonia, Finland, Germany, Greece, Italy, Latvia, Luxembourg, Slovenia, United States
	65+, every 10 years	France^b^, Lichtenstein^b^, Portugal^b^, Switzerland^c^
	Other	New Zealand (every 20 years), Slovakia (50+, every 15 years), Sweden (50+, every 20 years)
Tetanus	No national recommendation	Denmark, Hungary, Iceland, Ireland, Japan, Republic of Korea, Malta, Netherlands, Norway, Romania, United Kingdom
	Single booster	Croatia (at age 60), Poland (in adulthood), Spain (at age 65)
	Adult, every 10 years	Austria^a^, Belgium, Bulgaria, Canada, Cyprus, Estonia, Finland, Germany, Greece, Italy, Latvia, Luxembourg, Slovenia, United States
	65+, every 10 years	Czech Republic^c^, France^b^, Lichtenstein, Portugal^b^, Switzerland^c^
	Other	Lithuania (every 5–10 years), New Zealand (every 20 years), Slovakia (every 15 years), Sweden (every 20 years)
Pertussis	No national recommendation	Bulgaria, Canada, Croatia, Cyprus, Denmark, Estonia, Finland, Hungary, Iceland, Ireland, Latvia, Lithuania, Japan, Republic of Korea, Malta, Netherlands, New Zealand, Norway, Poland, Portugal, Romania, Slovakia, Spain, Sweden, Switzerland, United Kingdom
	Single booster in adulthood	Belgium, Czech Republic (at age 65), France, Germany, Greece, Slovenia, United States
	Adult, every 10 years	Austria^a^, Italy, Luxembourg
	65+, every 10 years	Lichtenstein^b^
Shingles (Herpes zoster)	No national recommendation	Bulgaria, Croatia, Cyprus, Denmark, Estonia, Finland, Germany, Hungary, Iceland, Ireland, Japan, Republic of Korea, Latvia, Lithuania, Luxembourg, Malta, New Zealand, Netherlands, Norway, Poland, Portugal, Romania, Slovakia, Slovenia, Spain, Sweden
	Vaccination at 50+	Austria, Czech Republic
	Vaccination at 60+	Canada, Greece, United States
	Vaccination at 65+	Belgium^d^, France^e^, Italy
	Vaccination at 70+	Australia, United Kingdom
Influenza (Trivalent)	No national recommendation	Sweden^f^
	Annual vaccination at 60+	Germany^f^, Greece^f^, Hungary, Iceland, Netherlands, Slovakia
	Annual vaccination at 65+	Australia, Belgium^f^, Bulgaria^f^, Canada, Croatia, Cyprus, Denmark, Estonia^f^, Finland^f^, France^f^, Ireland^f^, Italy^f^, Japan, Republic of Korea, Latvia^f^, Lithuania^f^, Luxembourg, New Zealand, Norway^f^, Portugal, Romania^f^, Spain^f^, Switzerland^f^, United Kingdom^f^
	All adults	Austria^f^, Czech Republic^f^, Malta^f^, Poland^f^, Slovenia^f^, United States^f^
Pneumonia (*S. pneumoniae*)	No national recommendation	Bulgaria, Croatia, Estonia, France, Latvia, Lichtenstein, Lithuania, Netherlands, New Zealand, Portugal, Romania, Switzerland^h^
	50+, PPV	Hungary
	50+, PCV	Poland
	50+, PCV and PPV^g^	Austria
	60+, PPV	Germany, Iceland
	60+, PCV	Slovakia
	60+, PCV and PPV^g^	Luxembourg
	65+, PPV	Australia, Canada, Cyprus, Ireland, Japan, Republic of Korea, Norway, Spain, Sweden, United Kingdom
	65+, PCV	Greece, Malta
	65+, PCV and PPV^g^	Belgium, Czech Republic, Italy, United States
	65+, PCV or PPV	Denmark, Finland, Slovenia

This table compiles recommended, age-specific vaccinations for older adults (from 50 years of age). It does not include catch-up vaccinations for vaccines typically given at younger ages, vaccines which are available but not recommended or reimbursed, vaccinations recommended for specific risk groups or vaccinations recommended in response to specific activities such as travel or transplantation

*PCV* pneumococcal conjugate vaccine, *PPV* pneumococcal polysaccharide vaccine

^a^Every 5 years from age 65

^b^Every 20 years for younger adults

^c^Every 10–15 years for younger adults

^d^Cap at 79 years of age

^e^Cap at 75 years of age

^f^Quadrivalent vaccine also available, though prioritization and access varies by region

^g^Initial dose is the conjugated pneumococcal vaccine, followed by the polysaccharide pneumococcal vaccine

^h^The use of PPV, with or without PCV, is being re-evaluated

### Adult vaccination: building effective programs

To understand why adult vaccination coverage is low, it is useful to examine those countries that have achieved the highest coverage and also to compare adult vaccination programs with pediatric vaccination programs. Successful pediatric vaccination programs generally recommend universal vaccination, they are supported by effective funding mechanisms, and the outcomes are assessed (and the programs corrected, if needed) based on routine surveillance of disease and vaccination coverage.

The important role of recommendations is suggested by the observation that countries with comprehensive vaccination recommendations for older adults tend to include more vaccines in their programs [68] and to reach higher levels of coverage with the recommended vaccines [67]. In addition, there is evidence that vaccination recommendations focusing on groups at risk, although apparently offering an efficient approach to vaccination, may actually inhibit uptake, since they may inadvertently send the message that the national health system does not see the recommended vaccines as important [79]. However, this evidence must be critically assessed in the light of economic evaluations, which often conclude that universal vaccination of older adults against a particular VPD is not cost-effective and that programs must be targeted to specific, well-characterized groups to ensure acceptable incremental costs per QALY gained [80].

As all the evidence identifies provider recommendation as the principal reason for adults to become vaccinated [1], this implies that if few providers are convinced of the importance of adult vaccination the uptake will remain low. Numerous studies show that many primary-care physicians do not consider vaccination of older adults a high priority [81–85].

Without funding, however, recommendations have limited effect. While most high-income countries have some form of public funding in place for recommended vaccines [82], cost can still be a barrier to access and may discourage HCPs from recommending the vaccine. As one example illustrating this, moving from a partial to a full subsidy of pneumococcal vaccination for older adults in Australia raised the uptake from 39 to 73% in patients attending a large public hospital [86]. Similarly, pediatric vaccination in the United States is financially supported by the Vaccines for Children initiative and this has virtually eliminated previously significant regional, ethnic and socioeconomic disparities in vaccination coverage [87]. In contrast, the same vaccines for adults can require substantial copayments, and ethnic and socioeconomic disparities in coverage rates are clearly visible [70].

**Fig. 2:**
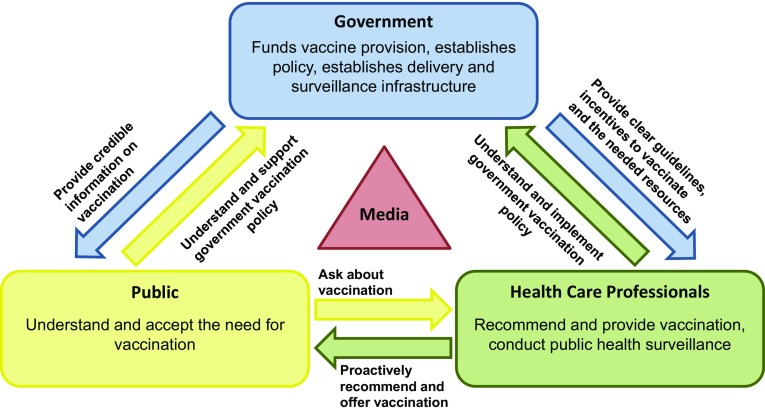
Components of successful vaccination programs. Healthcare systems are more than just government and business infrastructure: they comprise everyone involved in the process—politicians, healthcare providers and the general public. Each part of the polity engages in dialogue with the other parts, and it is plain that for the establishment of successful vaccination programs—whether in children or in adults—all parts must be in general agreement [88, 89]. In addition, each part of the polity has specific roles in terms of delivery, dialogue and acceptance, as indicated by the labeled arrows. The media plays a significant, but different role. While not (in theory) directly involved in the process, it is the channel through which much of the dialogue is conducted, and can also act as an “amplifier”—for example, increasing the visibility and impact of public concerns or hesitancy, or alternatively promoting vaccination by reporting on disease-related deaths or vaccine benefits

These findings suggest that four things are necessary to support effective vaccination programs, both in children and in adults (Fig. 2). First, a clear commitment to vaccination must be reflected in a coherent, comprehensive public policy. Second, a commitment to fund and deliver vaccines to the population is required, whether via predominantly public funds as in the United Kingdom and the Netherlands, or a mixture of public and private funds as in the United States. Third, effective surveillance of vaccination coverage and the burden of disease is required, so that goals can be set, priorities established, and the effectiveness of the program monitored and adjusted, if necessary. Finally, the safety and value of vaccination must be understood and appreciated both by the target population and by vaccinating healthcare professionals.

The first three of these factors can be implemented by public policy and the initial steps have already been taken in many countries. However, for adult vaccination programs to achieve the same kind of success as pediatric programs have, they need a similar degree of population acceptance. This is particularly an issue for adult vaccination because there is a widespread public perception that vaccination is not needed [90] and the decision of the individual to seek or accept vaccination is crucial. For infant vaccination, the messages are targeted at the parents. For adult vaccination, the vaccine recipients must be reached with a message that can convince them of the value and safety of vaccination directly to themselves or to their family. Additionally, it is important that vaccinating healthcare personnel understand the value of vaccination both for themselves and for patients.

These considerations are supported by interventions that have been associated with substantial improvements in adult vaccination coverage [88, 89]. Important common elements to improve coverage seem to be: clear national objectives and commitments; incentives for healthcare personnel to vaccinate; vaccination reimbursement systems; information and awareness campaigns; clear coverage objectives. However, even programs considered as highly successful often plateau at a suboptimal level of coverage [89]. These plateaus vary between vaccine types thereby indicating that vaccine-specific issues must be addressed as well.

## Conclusion

All over the world, in rich and developing countries alike, a demographic shift towards an aging population is underway. How we handle aging populations will have major economic and healthcare implications in the next few decades. Many infectious diseases inflict a disproportionate burden of disease in older adults and can contribute to the onset of frailty, but may be prevented or attenuated by vaccination. This implies that vaccination can serve as the third pillar of a strategy to support healthy aging, alongside healthy diet and exercise. However, the uptake of vaccination by the target population is generally low and must be substantially improved if the potential of vaccines to reduce the morbidity, mortality, loss of quality of life and healthcare costs caused by VPDs is to be realized [12]. The available evidence indicates that vaccination coverage in older adults can be considerably improved, although there is a need for further research into the generalizability of particular interventions to improve coverage.

## Electronic supplementary material

Below is the link to the electronic supplementary material.

Additional file 1. Focus on the patient (PDF 117 kb)

## Abbreviations

QALY: Quality-adjusted life-year
QoL: Quality of life
VPD: Vaccine preventable disease
HZ: Herpes zoster
