# Supplementary material for: Vaccination programs for older adults in an era of demographic change
Source: Eur Geriatr Med. 2018 Mar 19;9(3):289–300. doi: 10.1007/s41999-018-0040-8 (PMC5972173; doi:10.1007/s41999-018-0040-8)
Supplement: Supplementary file 1 — Additional file 1. Focus on the patient (PDF 117 kb) [file 41999_2018_40_MOESM1_ESM.pdf]

Focus on the Patient

What is the context?

- Older adults are more at-risk from many infectious diseases and have a higher chance of becoming severely ill when infected.
- Many infectious diseases can be prevented by existing vaccines. In some cases, vaccination does prevent infection and succeeds in attenuating the disease severity.
- However, for older adults the preventive potential of widespread vaccination is rarely achieved.
- As older adults become an increasing proportion of the global population, there is a need to improve vaccination coverage against these risks.

What is new?

- Burden of disease in older adults can contribute to frailty, and may be prevented or attenuated by vaccination.
- Vaccination may serve as a third pillar for healthy aging along with healthy diet and exercise .
- Rates of vaccination in the older adults are generally low. Morbidity, mortality, low quality of life and healthcare costs could be reduced through higher vaccination uptake.
- Effective vaccination programs relies on:
  - a coherent, comprehensive public policy
  - a commitment to fund and deliver vaccines to the population
  - an effective surveillance of vaccination coverage and the burden of disease
  - the safety and value of vaccination must be understood and appreciated both by the target population and by vaccinating healthcare professionals.

What is the impact?

- Dedicated programs can achieve substantial improvements in vaccination coverage in older adults and can be considerably improved.
- We need to do further research on how, or if, successful interventions in one place can be generalized to other places to improve coverage generally.
